# Psychobiotic Protection of Nutritional Supplements and Probiotics in Patients Undergoing Hemodialysis: A Randomized Trial

**DOI:** 10.3390/nu17040652

**Published:** 2025-02-12

**Authors:** Eric Climent, Francisco Hevilla, Marina Padial, Guillermina Barril-Cuadrado, María Blanca, Tamara Jiménez-Salcedo, Maria López-Picasso, Ángel Nogueira-Pérez, Gabriel Olveira

**Affiliations:** 1 Departamento de Biotecnología, Universitat Politècnica de València, 46022 Valencia, Spain; science@ericcliment.com; 2 Servicio de Endocrinología y Nutrición, Hospital Regional Universitario, 29071 Málaga, Spain; pako_hvs_296@hotmail.com (F.H.); mpadial27@gmail.com (M.P.); 3 Instituto de Investigación Biomédica de Málaga-Plataforma BIONAND, 29010 Málaga, Spain; 4 Departamento de Medicina y Dermatología, Universidad de Málaga, 29071 Málaga, Spain; 5 Servicio de Nefrología, Hospital la Princesa, 28006 Madrid, Spain; gbarril43@gmail.com (G.B.-C.); noro1976@hotmail.com (Á.N.-P.); 6 Servicio de Endocrinología y Nutrición, Hospital Rey Juan Carlos, 28933 Madrid, Spain; mblancamb@gmail.com (M.B.); maria.lopez@hospitalreyjuancarlos.es (M.L.-P.); 7 Servicio de Nefrología, Hospital Regional Universitario, 29010 Málaga, Spain; tajimsal@hotmail.com; 8 CIBER de Diabetes y Enfermedades Metabólicas Asociadas, Instituto de Salud Carlos III, 29071 Málaga, Spain

**Keywords:** microbiome, mental health, hemodialysis, probiotics, oral nutrition supplement

## Abstract

Background/Objectives: The prevalence of depression and anxiety symptoms is remarkably high in malnourished individuals undergoing hemodialysis. The goal of this project was to evaluate the impact of administering an oral nutritional supplement combined with a probiotic blend on the microbiota, intestinal permeability, and depression symptoms in malnourished hemodialysis patients. Methods: With this aim, a randomized trial was conducted with three parallel groups: a control group with individualized diet, a supplement–placebo (SU-PL) group with oral nutritional supplementation (ONS), and a supplement–probiotic (SU-PR) group with ONS in conjunction with a probiotic blend. Blood and fecal samples were collected at basal time, and at 3 and 6 months. Several blood biomarkers, like zonulin, lipopolysaccharide-binding protein (LBP), lipopolysaccharide (LPS), and brain-derived neurotrophic factor (BDNF), were measured, and the fecal microbiome was sequenced with the Illumina platform. The Hospital Anxiety and Depression Scale (HADS) was used for the estimation of depression (HADS-D) and anxiety (HADS-A) symptoms, along with the standardized mental health index SF12-MH from the general health questionnaire SF-12. Results: The results showed that patients who consumed the probiotic blend maintained the LPS levels from their baseline readings and decreased their BDNF levels compared to the SU-PL or control groups. Moreover, a significant decrease in HADS-D scores (less depressive symptoms) and an increase in SF12-MH scores (higher quality of life) were found in that group in comparison to the other groups. The intervention produced an impact on the microbiome population, where the SU-PR group had reduced *Akkermansia* abundance with respect to the other groups, while their *Acidaminococcus* abundance decreased and their *Barnesiella* abundance increased with respect to the SU-PL group. Conclusions: Overall, the results indicate that the probiotic with the nutritional supplement could reduce the intestinal permeability biomarkers and improve depressive symptoms and quality of life in malnourished hemodialysis patients.

## 1. Introduction

Chronic kidney disease (CKD) is characterized by the accumulation of uremic toxins, metabolites that the kidneys can no longer effectively eliminate [1]. Its global prevalence surpasses 10% of the general population, affecting more than 800 million individuals worldwide, with approximately one in every seven adults in Spain grappling with this condition [2]. Treatment primarily involves a combination of lifestyle adjustments, pharmaceutical interventions, and, in severe cases, medical procedures like renal replacement therapy, encompassing dialysis or kidney transplantation.

Renal replacement therapy is required by less than 1% of the population [2]. Among those undergoing dialysis, anxiety and depression emerge as the prevailing psychiatric comorbidities, with estimated prevalence rates ranging from 15% to 38% for anxiety and from 37% to 42% for depressive symptoms [3]. In addition, the presence of symptoms of depression and anxiety in this population is associated with the presence of malnutrition [4].

Treatment strategies span a spectrum of methodologies, including the utilization of antidepressant medications and cognitive behavioral therapy. Recent reviews have presented moderate-quality evidence underscoring the efficacy of cognitive behavioral therapy in alleviating depression among individuals undergoing dialysis. Conversely, the use of antidepressant medication remains constrained and inconclusive [5,6].

In recent times, there has been a growing interest in nutritional strategies, particularly those involving probiotics and psychobiotics, to address depression and anxiety. The identification and characterization of the gut–brain axis has transformed our comprehension of the microbiome’s impact on the human body, emphasizing the bidirectional communication between the brain and the gut [7]. This relationship extends beyond digestive disorders, influencing mental health as well.

Numerous theoretical mechanisms explain how the gut microbiota interacts with the central nervous system, potentially impacting the onset or amelioration of chronic diseases, as well as human behavior. These mechanisms encompass immunological (gut–immune system), biochemical, and neuroendocrine (HPA axis) pathways, alongside the metabolic processes of the gut microbiota, the integrity of the intestinal mucosal barrier, and the blood–brain barrier. In this intricate network of communication, the vagus nerve has emerged as a pivotal conduit [8].

Most psychiatric conditions have psychological, social, or biological determinants that can explain their origin. One of the key mechanisms influencing the biological factors of mental health is the modulation of the synthesis of neurotransmitters, such as serotonin and GABA, and the regulation of short-chain fatty acids (SCFAs), lipopolysaccharide (LPS) reduction, and inflammatory cytokines, as well as the endocrine system [9].

In recent years, there has been a growing interest in the use of probiotics in populations at risk of anxiety and depression. Primary tests have demonstrated the ability of certain bifidobacteria and lactobacilli to diminish inflammatory cytokines and oxidative stress, which may finally lead to increase brain-derived neurotrophic factor (BDNF) levels in the hippocampus, providing protection against anxiety and depressive-like behaviors [10,11].

In 2019, a pivotal “Renacare Trial” was carried out, involving a randomized, multicenter, double-blinded clinical study focused on malnourished hemodialysis patients. This trial featured the division of patients into three distinct groups, each subjected to a different intervention approach: (1) a control group receiving an individualized diet, (2) another group supplemented with oral nutritional supplements (ONSs) alongside a placebo, and (3) a third group receiving ONSs in conjunction with probiotics. The primary findings of this study indicated a notable increase in both body weight and fat-free mass (FFM), along with a reduction in inflammation/oxidation biomarkers [12].

This study constitutes a secondary analysis of the Renacare Trial. Given the high prevalence of psychiatric comorbidities among hemodialysis patients, mental health parameters were also measured during the original trial. This secondary analysis specifically aims to evaluate the impact of ONSs combined with probiotics on gut microbiota composition and anxiety and depressive symptoms in hemodialysis patients.

## 2. Materials and Methods

### 2.1. Design

The study design and details about the inclusion/exclusion criteria were reported in a previous publication [12]. We included malnourished adult subjects (>18 years) undergoing a hemodialysis therapy that was not modified in the 3 months before inclusion and for 6 months during the intervention. Written informed consent was obtained. The exclusion criteria included uncontrolled diabetes, severe organ disease, recent hospitalizations, gastrointestinal conditions, malignancy, pregnancy, prior supplementation (ONS, omega-3, probiotics), drug abuse, and concurrent participation in other studies. The enrolment took place in a hospital setting across three dialysis centers, ensuring close clinical monitoring and adherence to the study protocols.

A randomized, multicenter, parallel-group clinical trial with 3 groups was conducted—open regarding the intake of ONS or individualized diet recommendations, but double-blind for the intake of probiotics. Patients were randomized to one of the following three groups, using the study’s electronic data collection notebook, which ensured a 1:1 randomization ratio and maintained allocation concealment throughout the process:

1. Control (C): received individualized dietary recommendations.
2. ONS + placebo (SU-PL): received ONS and dietary recommendations.
3. ONS+ probiotics (SU-PR): received ONS with probiotics and dietary recommendations.

The etiology of chronic kidney disease and the duration of maintenance hemodialysis were considered during patient enrollment. The average time on hemodialysis was similar across the study groups: C (2.3 ± 1.1 years), SU-PL (3.3 ± 1.6 years), and SU-PR (2.6 ± 1.4 years), with no statistically significant differences (*p* = 0.443). Regarding the etiology of chronic kidney disease, the distribution was as follows: diabetes (C: 36.4%, SU-PL: 30%, SU-PR: 40%), nephroangiosclerosis (C: 27.2%, SU-PL: 30%, SU-PR: 20%), and other causes (C: 36.4%, SU-PL: 40%, SU-PR: 40%). No statistically significant differences were found between the groups (*p* = 0.98). These results indicate a comparable baseline profile regarding the kidney disease etiology and hemodialysis duration across the study groups.

The intervention lasted for 6 months. The Renacare ONS was specifically developed for malnourished hemodialysis patients. It is high in energy (2 kcal/mL) and proteins and enriched with functional nutrients (extra-virgin olive oil, omega-3 fatty acids, whey protein, antioxidants, low-glycemic-index carbohydrates, fiber, and carnitine) and soluble fiber (soluble/insoluble: 70%/30%): FOS, acacia fiber, and oat fiber. The nutritional composition is shown on the website https://adventiapharma.com/nutricion-clinica/productos/enteral-oral/bi1-renacare-dialysis/ (accessed on 20 January 2025).

The probiotics and the placebo were supplied as capsules indistinguishable by their external appearance (one capsule of 380 g). Each capsule of probiotic contained live bacteria: *Bifidobacterium breve* CNCM I-4035 (1.00 × 10^9^ colony-forming units (CFU)), *Bifidobacterium animalis lactis* CECT 8145 (3.50 × 10^9^ CFU), and *Lactobacillus paracasei* CNCM I-4034 (5.00 × 10^8^ CFU). *B. animalis* CECT 8145 was selected for its antioxidant properties and its metabolic pathways related to the metabolism of tryptophan, an essential amino acid that serves as the sole precursor for several neurotransmitters, such as serotonin [13]. This probiotic has also been used in a previous study with patients with Prader–Willi syndrome, resulting in a reduction in anxiety symptoms [14]. *L. paracasei* CNCM I-4034 and *B. breve* CNCM I-4035 were selected for their immunomodulatory effects, which have been previously demonstrated [15].

### 2.2. Outcomes

All procedures were performed at baseline and after 3 and 6 months.

#### 2.2.1. Blood Biomarkers

Fasting blood samples were drawn before the dialysis session; plasma and serum were separated into aliquots and stored until analysis at −80 °C in the Hospital-IBIMA biobank, which also belongs to the Andalusian Public Health System Biobank, belonging to the National Biobank Platform (exp. PT20/00101). The serum levels of gut permeability biomarkers were determined by enzyme immunoassay techniques, following the manufacturer’s instructions: lipopolysaccharide-binding protein (LBP) (MyBioSource Inc., San Diego, CA, USA; intra-assay CV ≤ 8%; inter-assay CV ≤ 10%), zonulin (Wuhan Huamei Biotech Co., Wuhan, China; intra-assay CV ≤ 8%; inter-assay CV ≤ 10%), and lipopolysaccharides (LPSs) (Wuhan Huamei Biotech Co., Wuhan, China; intra-assay CV ≤ 8%; inter-assay CV ≤ 10%). Other inflammation and oxidation biomarkers, such as pro-inflammatory cytokines and atherosclerosis biomarkers, were measured and compared between groups and are included in the previous publication [12].

#### 2.2.2. Psychological Questionnaires

The SF-12.v2 Spain Questionnaire was used. It consists of twelve items with two final dimensions measuring physical health (SF12-PH: Standardized physical health component scale) and mental health (SF12-MH: Standardized mental health component scale). The score ranges from 0 to 100, where a higher score implies a better health-related quality of life (https://www.bibliopro.org/buscador/663/cuestionario-de-salud-sf-12 (accessed on 20 January 2025)). Symptoms of anxiety and depression were measured with the Hospital Anxiety and Depression Scale (HADS), a self-assessment scale that is valid for screening and reports patients’ symptoms in the past week. This questionnaire is divided into two independent subscales: anxiety (HADS-A) and depression (HADS-D). A score of 0 to 7 for either subscale is considered normal, a score of 8 to 10 is suggestive of the presence of the mood disorder, and score of 11 or higher indicates probable presence of the respective state [16].

#### 2.2.3. DNA Sequencing

Fecal samples were collected in DNA preservation solution and kept at −80 °C until their arrival at sequencing facilities. Highly pure DNA was extracted from the samples using the Qiasymphony protocol with the PowerFecal Pro kit (QIAgen, Valencia, CA, USA), that combines enzymatic and mechanical lysis. Genomic libraries were constructed using PCR products obtained from the 16S rRNA gene’s V3–V4 region [17]. DNA libraries were sequenced with the Illumina MiSeq 500 sequencer (Illumina Inc., San Diego, CA, USA), using a paired-end 300-nucleotide strategy.

#### 2.2.4. Bioinformatics Analysis

Paired-end reads were merged using the bbtools toolkit [18]. Then, they were trimmed in order to remove the PCR primers with cutadapt v3.7 software [19]. After that, sequences whose quality score remained under 20 on the Phred scale were removed from the analysis. DADA2 v1.26 software [20] was used to denoise samples, remove chimeric sequences, and make ASVs (amplicon sequence variants). Taxonomic annotation was conducted following a mixed strategy, using the NCBI 16S and SILVA databases [21]. NCBI annotation was carried out using BLAST v2.15.0+ software, and hits with identity scores under 97% were annotated with the SILVA database and the nbayes algorithm from Qiime2 [22]. With this strategy, 80% of ASVs were unambiguously annotated at the genus level, and 85% arrived at the family level.

#### 2.2.5. Statistical Analysis

Data analysis was carried out using R software version 4.1.3. Between-group comparisons were tested with parametric (T-test) and non-parametric (Wilcoxon) tests, depending on the normality of the data. To compare variables according to the group and time (baseline, 3 months, and 6 months), ANOVA for repeated variables was used when the normality assumption was fulfilled. Otherwise, the necessary non-parametric techniques were applied: intra-subject Friedman (post hoc Wilcoxon) and inter-subject Kruskal–Wallis H (post hoc Mann–Whitney U) tests. Linear mixed models were also used to check differences and interactions between groups and visits on the clinical parameters, with the R package lmer. The level of significance considered was 5%, and for multiple comparisons (post hoc), Bonferroni correction was considered. Differential taxonomy analysis was conducted with the DESeq2 library [23], using a complete model (~Intervention + Time + Intervention:Time), and differences were checked with the Wald test. Associations between microbiome and blood metabolites and mental health parameters were also measured with DESeq2, scaling and centering the numerical values.

## 3. Results

**Figure 1:**
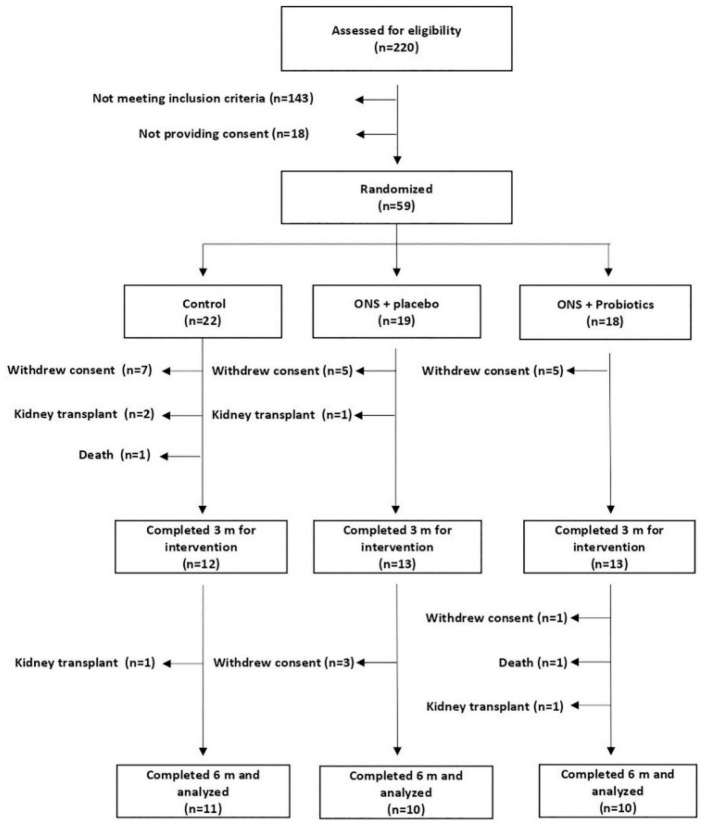
Flow diagram outlining the allocation of the patients to the different arms of the trial.

A total of 31 patients (11 corresponding to group C, 10 to SU-PL, and 10 to SU-PR) completed the 6-month trial (Figure 1) [12].

### 3.1. Effects on Clinical Parameters by Group

At the baseline visit, there were no basal significant differences between groups regarding age, sex, diabetes, Charlson comorbidity index, or intake of fermented milk or antibiotics during the month prior to inclusion (Table 1). Furthermore, there were no baseline differences in any of the parameters for the nutritional assessment, dietary intake, or biochemical data [12].

**Figure 2:**
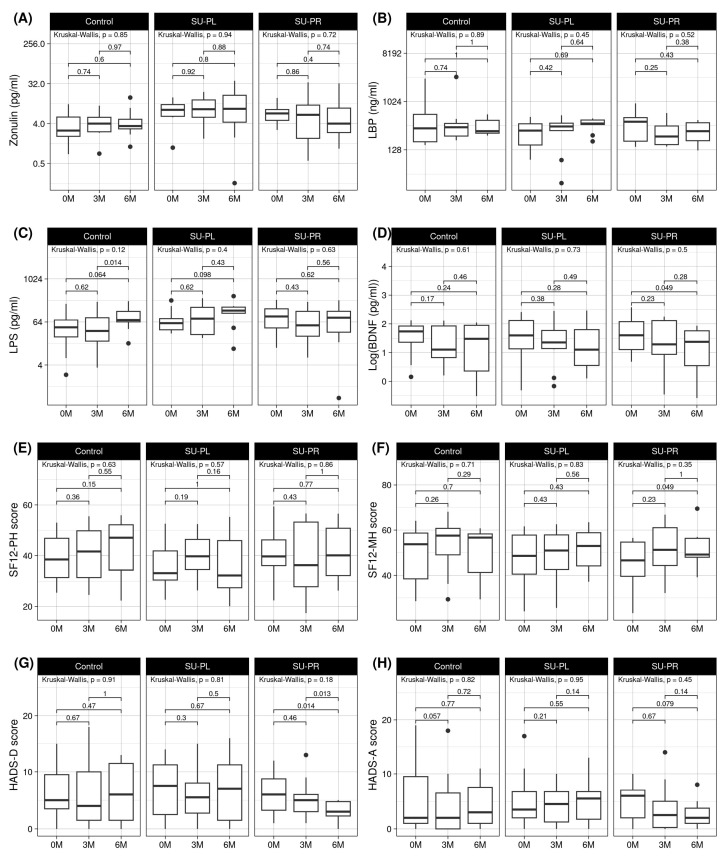
Gut permeability biomarkers: (**A**) zonulin, (**B**) LBP (lipopolysaccharide-binding protein), (**C**) LPS (lipopolysaccharide), (**D**) logarithm of BDNF (brain-derived neurotrophic factor), (**E**) HADS-D score (depression score of the Hospital Anxiety and Depression Scale), (**F**) HADS-A score (anxiety score of the Hospital Anxiety and Depression Scale), (**G**) SF12-PH score (physical health score of the 12-item Short Form), and (**H**) SF12-MH score (mental health score of the 12-item Short Form) across visits in the three different intervention groups. Values beyond 1.5 times the interquartile range are plotted as individual points.

The statistical analyses showed no significant differences between groups in LBP and zonulin measurements along the three timepoints. LPS levels increased in the C group (*p* = 0.014), whereas SU-PL showed a trend of increase (*p* = 0.098), and the SU-PR group maintained the basal levels (Figure 2). Differences between groups were also tested with a linear mixed model, with significant results on the interaction of the SU-PR group and 6-month visit, compared to the control group (*p* = 0.007), supporting the idea that the SU-PR group behaved differently than the control and SU-PL groups. Interestingly, brain-derived neurotrophic factor (BDNF) was reduced significantly in the SU-PR group (*p* = 0.049) (Figure 2).

Moreover, pro-inflammatory factors were measured in serum, such as monocyte chemoattractant protein-1 (MCP-1), interleukin-4, and total antioxidant capacity (TAC), among others, finding significant decreases in supplemented patients, especially in the group containing probiotics (results previously reported in [12]).

### 3.2. Effects on Mental Health Parameters by Group

At the baseline visit, there were no differences in any of the scales assessed. Comparative analysis showed that while SF12-PH did not show significant differences, the SF12-MH score increased significantly at 6 months in the SU-PR group, while in the C and SU-PL groups it remained stable (Figure 2) (Supplementary Table S1). The results of the HADS-D questionnaire showed similar figures, with a significant decrease in the depression-associated score in subjects who consumed the probiotic, compared to the other groups. At the sixth month, all subjects who had received the probiotic had normal values of HADS-D, compared to basal time, when only 50% of subjects were in the normal category of HADS-D. The Wilcoxon test confirmed that the HADS-D values decreased over time in the SU-PR group (*p* = 0.013), and the linear mixed model identified that this decrease was only significant in the interaction between the SU-PR group and the 6-month visit (*p* = 0.03), compared to the control. Regarding the HADS-A index, no significant changes were observed, but a decreasing trend was present in the SU-PR group (*p* = 0.079) (Figure 2) (Supplementary Table S1).

### 3.3. Effects on Gut Microbiota and Their Association with Clinical Outcomes

Amplicon sequencing was performed on the 93 stool samples, resulting in an average of 101,800 sequences per sample. The most abundant phylum was Firmicutes (60.88%), followed by Bacteroidota (23.87%), Actinobacteriota (7.62%), and Proteobacteria (4.65%).

**Figure 3:**
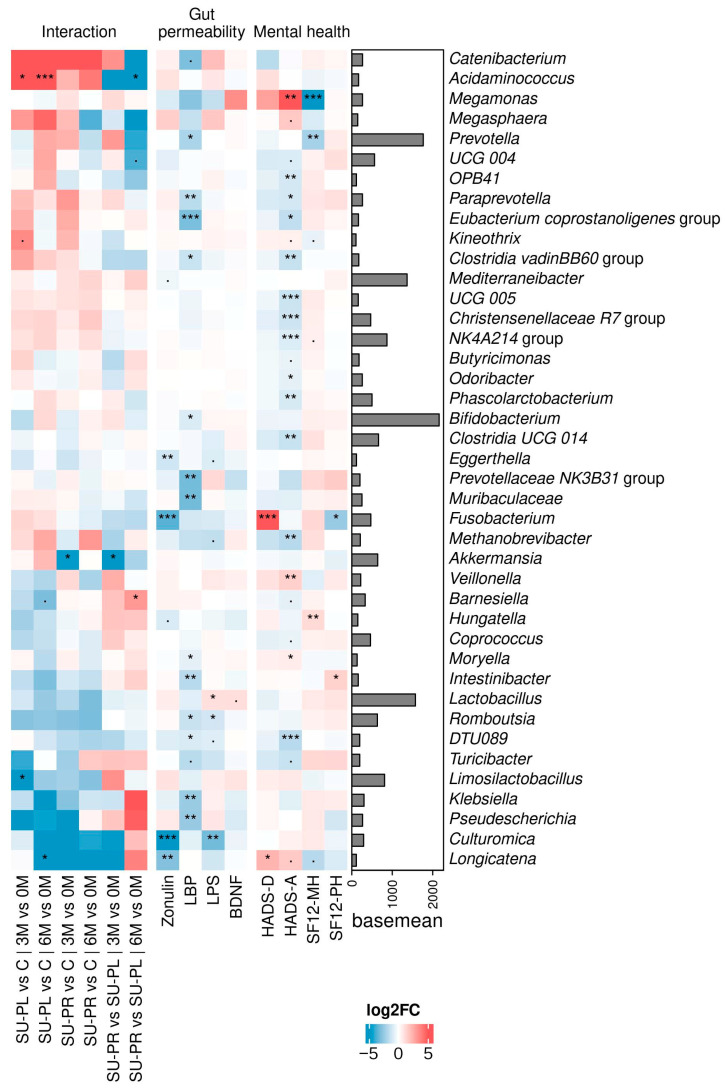
DESeq2 correlation analyses between patient clinical measures and fecal bacterial abundance. Left: heatmap showing the log fold change (Log2FC) of genus abundance, comparing the interaction of different groups depending on the time. Positive values of log2FC in the interaction results correspond to greater increase in the first group, during the time interval shown. The average presence of each genus across all samples is shown with a bar plot. (· = *p* < 0.1, * = *p* < 0.05, ** = *p* < 0.01, *** = *p* < 0.001).

Alpha and beta diversity were calculated during the intervention, resulting in no significant changes between groups (Supplementary Figure S1). The fecal microbiota composition changed differently between groups across the three timepoints (Figure 3). The abundance of *Limosilactobacillus* and *Longicatena* increased more in the control group than in the SU-PL group, when comparing the visits at 3 and 6 months to the first visit, respectively, while *Acidaminococcus* decreased over time in the control group compared to the SU-PL group.

At month 3, only the *Akkermansia* genus presented differences when SU-PR was compared with both control and SU-PL over time, with its abundance being lower in the SU-PR group than in the control and SU-PL groups. At month 6, the abundance of *Acidaminococcus* decreased compared to the SU-PL group, while *Barnesiella* had a significant increase in the same period.

Subsequently, we conducted correlation analyses between the bacterial profile and several clinical parameters, like zonulin, LBP, LPS, and BDNF. In terms of gut inflammation biomarkers, we observed significant negative correlations between zonulin and the abundance of the *Culturomica*, *Eggerthella*, *Longicatena*, and *Fusobacterium* genera. Likewise, LBP was negatively correlated with the genera *Bifidobacterium*, *Prevotella*, *Romboutsia*, *Paraprevotella*, *Klebsiella*, *Eubacterium coprostanoligenes* group, *Moryella*, *Muribaculaceae*, and *Pseudescherichia*, sorted by decreasing abundance. Additionally, LPS showed significant negative correlations with the *Romboutsia* and *Culturomica* genera and was positively correlated with *Lactobacillus* genus abundance. Moreover, BDNF showed a positive correlation (*p* = 0.09) with *Lactobacillus* abundance.

Turning our attention to psychological parameters, SF12-PH displayed a significant negative correlation with *Fusobacterium* and a positive correlation with *Intestinibacter*. In contrast, MCS12 was negatively correlated with *Megamonas* and *Prevotella* abundances, while showing a positive correlation with *Hungatella*. The HADS-D score was positively correlated with *Fusobacterium* and *Longicatena* abundances. Notably, the HADS-A index demonstrated a higher number of correlations compared to the other indices. We identified a significant negative correlation with *Paraprevotella*, *Odoribacter*, *Clostridia*, and *Methanobrevibacter* abundances, and positive correlations with *Megamonas* and *Veillonella* abundances.

## 4. Discussion

As far as our knowledge extends, this study represents the first attempt to explore the effects of probiotic supplementation on the mental well-being of hemodialysis patients and their microbiome correlation, employing advanced next-generation sequencing techniques. To achieve our objective, we conducted a comprehensive analysis of mental health metrics and the microbiome profiles of participants enrolled in the randomized RENACARE trial. This trial featured three parallel groups: a control group following an individualized diet, a placebo group receiving an oral nutritional supplement (ONS) alongside a probiotic blend designed to modulate the microbiota, and a group administered the ONS in conjunction with a probiotic blend.

Our findings indicate an enhancement in the mental health quality of life index (SF12-MH) and a reduction in the depression subscale (HADS-D) among patients whose HADS-D was over 8 points, and who underwent intervention with the probiotic blend and ONS (SU-PR). This was accompanied by reductions in lipopolysaccharide (LPS) levels and a modulation of the gut microbiome. Moreover, our findings restate the reduction in brain-derived neurotrophic factor (BDNF) that was already described in previous analyses [12].

Differential variations in the microbiota were detected in those volunteers who consumed the dietary supplement and together with an added probiotic (SU-PR). The genera *Akkermansia*, *Acidaminococcus*, and *Barnesiella* were found in less abundance when individuals consumed the supplement with the probiotic added. These bacteria have been ascribed psychobiotic effects, and they also showed correlations with numerous physical and psychological parameters that were assessed in [24,25].

The main taxonomic difference between the SU-PR group and the control group was a decrease in *Akkermansia* at the 3-month visit. *Akkermansia* is a bacterium known for its ability to degrade intestinal mucin, and it is a regular inhabitant of the human intestine [26]. Recently, the positive effect of *Akkermansia muciniphila* (a majority species from the genus *Akkermansia*) in a variety of neuropsychiatric disorders has been put into question. This species was also reported to participate in the development of neuropsychiatric disorders by aggravating inflammation and influencing mucus production [27]. In low-fiber diets, *A. muciniphila* presents the ability to degrade the mucin of the intestinal mucus barrier, leading to an increase in intestinal permeability and inducing inflammatory responses, neurotoxicity, blood–brain barrier disruption, and pathogen susceptibility [28]. Also, its abundance is increased in brain diseases like multiple sclerosis [29], Parkinson’s disease [30], and depression [31] The reduction in *Akkermansia* abundance in the SU-PR group may be explained by the modulation effect on the microbiome induced by the probiotics, helping to maintain a controlled population of mucin-degrading bacteria.

Another group of interesting bacteria is the *Acidaminococcus* genus, which has been found to be overrepresented in several studies examining the relationship between the intestinal microbiota and schizophrenia [25]. This proteobacterial group from the *Clostridioides* family was increased in the SU-PL group with respect to the SU-PR group. In the same comparison, the genus *Barnesiella* increased with the probiotic. The *Barnesiella* genus is considered beneficial due to its ability to limit the colonization of pathogenic bacteria [32], and it is reduced in animal models of stress-induced conditions [33].

Furthermore, it is relevant to study the key bacteria that show significant correlations with the studied psychological indicators, to be able to identify potential biomarkers related to depression and anxiety. Some bacterial genera, such as *Fusobacterium*, *Veillonella*, *Megamonas*, or *Longicatena*, have been positively associated with the measured health indicators, indicating that these bacteria are increased in individuals with depressive and anxious traits. The abundance of both *Fusobacterium* and *Veillonella* has been associated with depressive disorders [34,35], while *Megamonas* shows contradictory results [36] and *Longicatena,* to the best of our knowledge, has not been associated with mental conditions yet.

On the other hand, taxa at the genus level, like *Clostridia UCG* 014, *Ruminococcaceae DTU089*, *Ruminococcaceae NK4A214* group, *Lachnospiraceae UCG 005*, *Phascolarctobacterium*, *Odoribacter*, or *Paraprevotella*, are negatively correlated with the HADS-A indicator, suggesting their overrepresentation in individuals with lower anxiety levels. Most of these genera have previously been negatively associated with major depressive disorder [37]. In the case of bacteria belonging to the *Lachnospiraceae* and *Ruminococcaceae* families, a reduction in abundance was observed in a rat model of depression compared to the control group. This reduction was strongly correlated with the host’s glycerophospholipid metabolism, which plays a significant role in the development of depressive and anxiety-like behavior [38,39].

Regarding the biomarkers that were measured in blood, LBP is a human protein, produced in hepatocytes, that binds to LPS in the outer membrane of Gram-negative bacteria. LPS is considered to be an endotoxin that can produce endotoxemia when released in the blood. For this reason, LBP performs an important function in the innate immune system, enhancing the response to infectious bacteria [40]. Concentrations of LBP in serum can increase in people with metabolic disorders such as obesity or diabetes. This increase is directly related to gut permeability to LPS and the subsequent rise in circulating LPS [41]. LBP is inversely correlated with the *Bifidobacterium* genus, indicating reduced intestinal inflammation in its presence. *Bifidobacterium* was part of the probiotic mix used in the SU-PL intervention, and its population increased in this group, but only at the 6-month visit. It remained stable in all three groups at the 3-month visit. This genus can capture LPS from different bacteria, such as *Escherichia* [42], which would explain its relationship with the levels of plasma LBP.

Furthermore, brain-derived neurotrophic factor (BDNF) is the most prominent neurotrophin associated with depression, playing a critical role in neural plasticity mechanisms [43]. Brain measures of BDNF are directly associated with lower levels of depression, but the same measures in plasma show an inverse correlation, suggesting that the effect of this biomarker may be dependent of its location, and highlighting the need for further studies to better understand the correlation of this biomarker with depression. The level of serum BDNF was reduced significantly in the SU-PR group. In addition, BDNF was positively correlated with *Lactobacillus*. The relationship between certain *Lactobacillus* strains and BDNF has already been stated [44] in animal models, where the supplementation with this probiotic genus increased the expression of BDNF and other brain-related factors. With these correlations, the role of *Bifidobacterium* and *Lactobacillus* in the human gut–brain axis is reinforced, emphasizing the need for further research in this area.

While the probiotic blend in combination with ONS administration holds the potential to play a role in depression symptoms and associated inflammation markers, the precise underlying mechanism remains unclear. We did observe a modulation of the microbiome; however, the genera with significant changes in the intervention did not exhibit a significant correlation with the clinical outcomes. This could be attributed to microbiome modifications occurring at a taxonomic level that cannot be adequately analyzed using the 16S technology approach. Furthermore, additional analyses need to be conducted to identify the underlying mechanism of action of the probiotic blend employed. This study presents several noteworthy limitations and strengths. One of the primary limitations lies in the relatively small sample size, which may impact the generalizability of the findings to a broader population. A greater sample size would have allowed for better stratification of the patients based on depression severity, dialysis vintage, and comorbidities, enabling the identification of subgroups that might benefit more from the treatment. Despite this limitation, this study boasts numerous strengths, including a comprehensive six-month follow-up period, active monitoring by trained dietitians, a multicentric design ensuring diverse patient inclusion, and meticulous assessment of both microbiota and biomarkers. These strengths provide a solid foundation for future investigations seeking to unravel the connections between probiotics, the gut microbiome, and clinical outcomes in the context of depression and inflammation.

Caution is advised when interpreting the results of this study, as the limited sample size of patients examined (partly due to the SARS-CoV-2 pandemic, the high dropout rate, and the associated difficulties in completing patient follow-ups) may affect the generalizability and robustness of the findings. Given the small number of subjects per group, their characteristics may differ more than expected. For example, the age difference between the control and intervention groups could have influenced some outcomes, particularly those related to gut microbiome composition, which is known to be age-sensitive. However, a statistical test was conducted to assess these potential differences, and no significant differences were found. Nonetheless, the statistical power for the variables that reached significance was above 80% in all cases. Furthermore, due to the original experimental design, the probiotic mix was only tested in combination with ONT, so the effects of the probiotics alone could not be discerned. A fourth study arm with only probiotic supplementation would help to understand their effects. Finally, analysis of fecal metabolites was lacking, and the intrinsic mechanisms of how the probiotics regulate were unknown. Future studies should expand the biomarker panel to include a more comprehensive range of inflammation markers (e.g., IL-6, TNF-α, and CRP), neurotransmitters (e.g., serotonin, dopamine, and GABA), and gut–brain axis mediators (e.g., short-chain fatty acids, mucin levels, and bacterial metabolites) to provide a more holistic understanding of the intervention’s effects. However, in previous publications, we have suggested possible mechanisms based on blood biomarkers [12]. As strengths of this study, we highlight that it is a randomized clinical trial (double-blind regarding probiotic intake) with long-term follow-up (6 months) and a multicenter design.

## 5. Conclusions

In conclusion, this study provides valuable insights into the potential impact of dietary and probiotic interventions on the microbiome of dialysis patients and their associated psychological and physiological parameters. We observed modest yet significant changes in key bacterial genera, which are known to play crucial roles in intestinal health and the gut–brain axis. Furthermore, this study reveals intriguing connections between microbiome composition and blood biomarkers like LBP and BDNF, emphasizing the bidirectional communication between the gut and the brain. These findings underscore the need for continued research in the emerging field of the gut–brain axis to unravel the intricate mechanisms through which the gut microbiota influences mental health and systemic physiology. The complex interplay between the gut microbiome and human health remains an exciting and promising area of exploration, with far-reaching implications for the fields of medicine and psychology.

## Figures and Tables

**Table 1:** Baseline characteristics the 31 patients enrolled in the study, divided by intervention arm. Differences between groups were not significant (ns).

	Control	SU-PL	SU-PR	*p*-Values
	(*n* = 11)	(*n* = 10)	(*n* = 10)	
Age (years) m ± ds	76.3 ± 8.7	65.1 ± 18.4	66 ± 18.5	ns
Sex, women % (*n*)	27 (3)	20 (2)	30 (3)	ns
Diabetes mellitus % (*n*)	36.4 (4)	40 (4)	30 (3)	ns
Antibiotic treatment in the last month	9.1 (1)	20 (2)	10 (1)	ns
Consumption of yogurt or fermented milk in the last month % (*n*)	63.6 (7)	60 (6)	60 (6)	ns
Charlson comorbidity index. m ± ds	4 ± 2.31	5.1 ± 2.02	4.18 ± 2.6	ns

## Data Availability

The data supporting this study’s findings have been deposited in the European Nucleotide Archive (ENA) at EMBL-EBI under accession number PRJEB75232.

## Supplementary Materials

The following supporting material can be downloaded at https://www.mdpi.com/article/10.3390/nu17040652/s1: Figure S1: Alpha and beta diversity. Table S1: Clinical measurements.
