# Supplementary figures and images for: Psychobiotic Protection of Nutritional Supplements and Probiotics in Patients Undergoing Hemodialysis: A Randomized Trial
Source: Nutrients. 2025 Feb 12;17(4):652. doi: 10.3390/nu17040652 (PMC11858206; doi:10.3390/nu17040652)

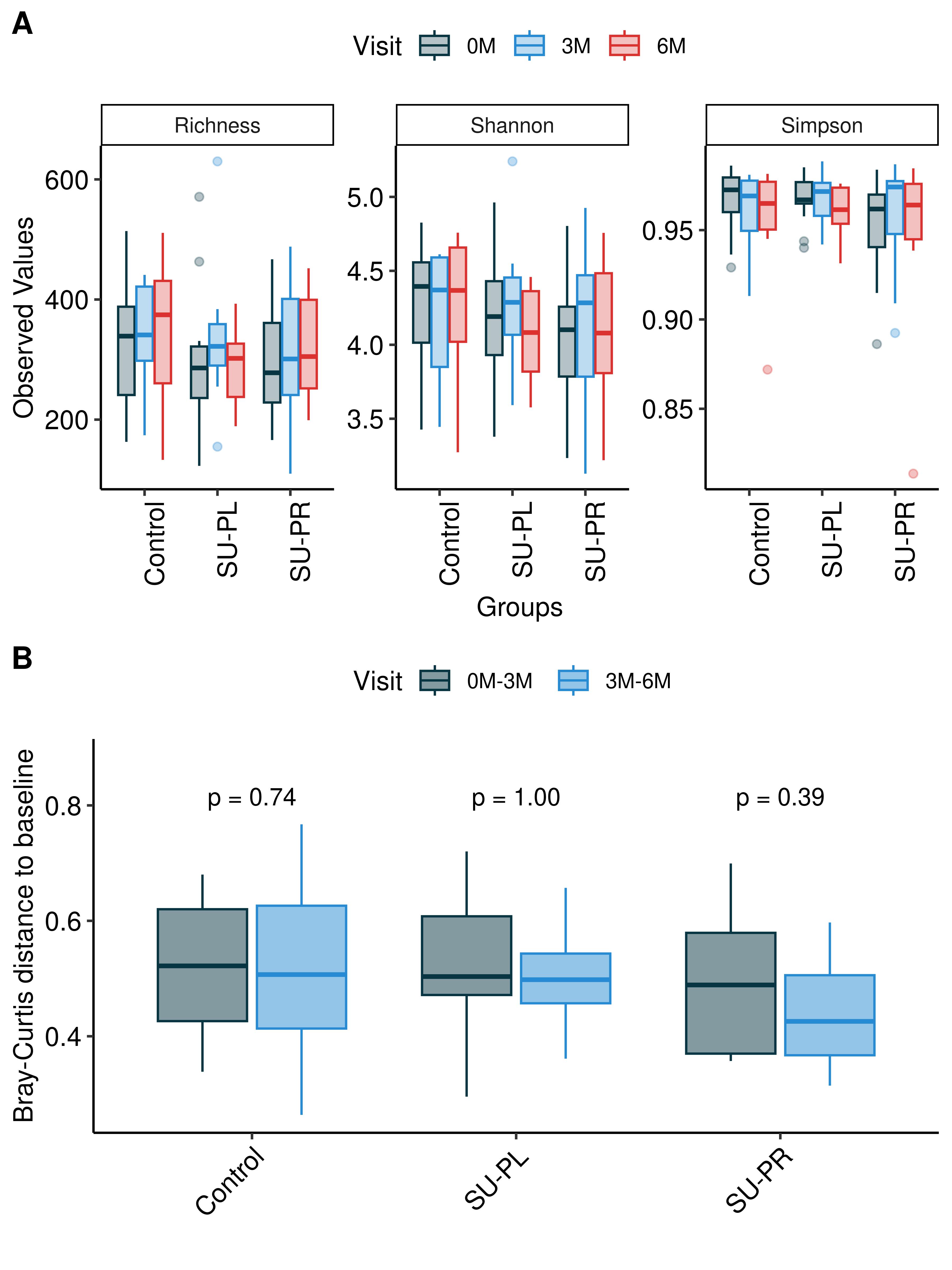

Supplement: Supplementary file 1 [file nutrients-17-00652-s001.zip › Supplementary_Figure S1.jpg]
